# ﻿Morphological and molecular evidence uncovers hidden species diversity in the leatherleaf slug genus *Valiguna* (Systellommatophora, Veronicellidae) from Thailand

**DOI:** 10.3897/zookeys.1212.126624

**Published:** 2024-09-13

**Authors:** Bowornluk Mitchueachart, Chirasak Sutcharit, Piyoros Tongkerd, Somsak Panha

**Affiliations:** 1 Biological Sciences Program, Faculty of Science, Chulalongkorn University, Bangkok 10330, Thailand; 2 Animal Systematics Research Unit, Department of Biology, Faculty of Science, Chulalongkorn University, Bangkok 10330, Thailand; 3 Academy of Science, The Royal Society of Thailand, Bangkok 10300, Thailand

**Keywords:** Anatomy, barcoding, COI gene, Indochina, pulmonate, systematics, taxonomy

## Abstract

The poorly studied leatherleaf slug genus *Valiguna* in Thailand was carefully investigated. Members of this genus are phenotypically similar, making their identification very challenging. This study clarifies the taxonomic status of all *Valiguna* species in Thailand by combining morphological and anatomical studies with DNA barcoding. Monophyly of all *Valiguna* species was confirmed by analysis of the mitochondrial COI data and that all *Valiguna* species have the acropleurocaulis type of penis. Currently, three *Valiguna* species are recognised: *V.siamensis*, *V.semicerina* Mitchueachart & Panha, **sp. nov.**, and *V.crispa* Mitchueachart & Panha, **sp. nov.** that are new to science. For distinct characteristics, *V.siamensis* is characterised by having a cylindrical penis and honeycomb-like glans, *V.semicerina***sp. nov.** has a lanceolate penis with half honeycomb-like glans, and *V.crispa***sp. nov.** has a cylindrical penis with wavy-like glans. In addition, more detailed descriptions of the radula and genitalia of all three species and their distribution are also carefully presented, enhancing the understanding of this leatherleaf slug genus in Thailand.

## ﻿Introduction

Land slug is a broad term (common name) used to describe terrestrial gastropods that either lack an external shell or possess an internal shell in the form of a flat plate or calcareous granules (Pearce and Örstan 2006). Through the process of limacisation, land slugs have independently convergently evolved within various Geophila families, with the majority of them belonging to the stylommatophorans (e.g., various arionoids, limacoids, and helicarionoids families), while lower numbers of them are found in the systellommatophorans (e.g., onchidiids, veronicellids, and rathouisiids) (Solem 1974; Hausdorf 2001; Cameron 2016). Additionally, land slugs have also evolved within non-Geophila families, such as *Aitengmarefugitus* Kano, Neusser, Fukumori, Jörger & Schrödl, 2015 (Kano et al. 2015). Generally, land slugs play various functions and essential roles in the ecosystem, such as prey, predators, detritivores, and vectors for the dispersal of plant seeds and soil invertebrates (Nyffeler and Symondson 2001; Türke et al. 2018). Some land slug species have been reported as invasive and agricultural pests (Naranjo-García et al. 2007; Sommer and Cowie 2020). Moreover, several land slug species were reported as intermediate hosts of *Angiostrongylus* spp. that cause zoonotic nematode infections in humans (Valente et al. 2020; Thiengo et al. 2022).

Veronicellidae Gray, 1840, known as ‘leatherleaf slugs’, belong to the systellommatophoran group, which can be easily recognised by having a flat body, dorsal (notum) covered by a thick mantle, ventral (hyponotum) with a narrow foot inside the pedal groove, and two pairs of tentacles: upper contractile tentacles and lower bifid tentacles (Gray 1840; Schilthuizen and Liew 2008). They are herbivorous, feeding on fresh and decaying plants, and can be found in both anthropogenic and natural habitats under tree logs, leaves, and other objects on the ground (Barker 2001). Currently, around a hundred species from 23 genera are distributed throughout the tropical and subtropical regions, and among these, around five species of three genera, *Filicaulis* Simroth, 1913, *Semperula* Grimpe & Hoffmann, 1924, and *Valiguna* Grimpe & Hoffmann, 1925, are recorded in Thailand (Simroth 1913; Grimpe and Hoffmann 1925a, 1925b; Hoffmann 1925; Thomé 1975; Thomé et al. 1994; Gomes and Thomé 2004; Gomes 2007; Gomes et al. 2008). These three genera have a very similar external morphology (body colour) but primarily differ in their reproductive organs: *Filicaulis* has an acrocaulis type of penis, *Semperula* has a pleurocaulis type of penis, and *Valiguna* has an acropleurocaulis type of penis (Grimpe and Hoffmann 1924; 1925a, 1925b; Gomes and Thomé 2004; Gomes 2007). Even though Thailand is known for having a high diversity of land snails (Naggs et al. 2006), no systematic and molecular works have been conducted on these veronicellid slugs other than the species list without a taxonomic description (i.e., Hemmen and Hemmen 2001; Sutcharit and Panha 2008).

*Valiguna*, the most common leatherleaf slug genus in Thailand, has been poorly understood and has remained unclear for several decades because the original descriptions frequently lack essential anatomical details, and several species are known from only a few specimens. The precise classification of these slugs is currently based on the penis and vas deferens morphology (Grimpe and Hoffmann 1925a, 1925b; Thomé 1984; Gomes 2007; Gomes et al. 2008). To address these challenges, a combined molecular phylogeny, particularly the cytochrome oxidase subunit I (COI or barcoding) gene, and morphological study (external characters, genitalia, and radula) was employed herein to assist in the systematic classification of the *Valiguna* in Thailand. This study is the first molecular work on the veronicellid slugs in Thailand, and includes a redescription for one species, and two new species are carefully described herein.

## ﻿Materials and methods

### ﻿Specimen sampling and morphological studies

**Figure 1.**
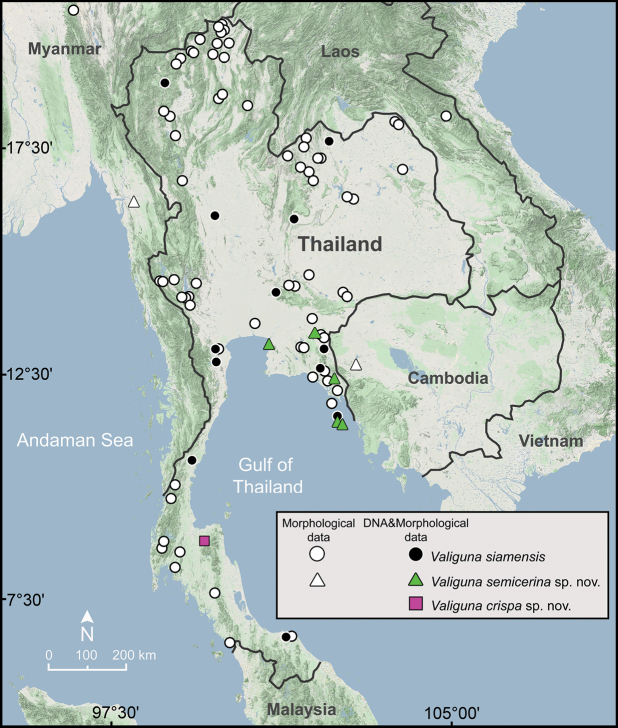
Geographic distribution of *Valigunasiamensis*, *V.semicerina* sp. nov., and *V.crispa* sp. nov. based on the specimens examined herein.

The 126 veronicellid specimens were sampled from limestone and non-limestone areas throughout Thailand (Fig. 1) and voucher specimens have been deposited in the Chulalongkorn University Museum of Zoology (**CUMZ**), Bangkok.

The living specimens were collected from litter on the soil surface and rotten logs, then photographed and euthanised by the two-step method (American Veterinary Medical Association, 2020). Specimens were preserved in 70% (v/v) ethanol for the morphological study and 95% (v/v) ethanol for the molecular study. The body length, body width, and foot width of specimens were measured using a ruler. Morphological examination was made under a stereo microscope with species identification based on the taxonomic literature (Martens 1867; Grimpe and Hoffmann 1925a, 1925b; Hoffmann 1925, 1941; Gomes and Thomé 2004; Gomes 2007; Gomes et al. 2008). Radulae were extracted and soaked in 10% (w/v) sodium hydroxide, rinsed with distilled water, and then examined using scanning electron microscopy (SEM; JEOL, JSM-6610 LV). The radula shape and teeth formula were investigated.

In the material part, the Thai terms “Tham” meaning cave, “Wat” meaning temple, “Khao” for mountain or hill, and “Koh” meaning island are used throughout for the locality names.

### ﻿Phylogenetic (COI) analyses

Total DNA was extracted from a piece of the foot using the NucleoSpin Tissue Kit (Macherey-Nagel, Germany), following the standard manufacturer’s procedure. A fragment of the mitochondrial COI gene was amplified by polymerase chain rection (PCR) using the universal primers LCO1490 (5′-GGTCAACAAATCATAAAGATATTGG-3′) and HCO2198 (5′-TAAACTTCAGGGTGACCAAAAAATCA-3′) (Folmer et al. 1994). Each PCR amplification (30 μL) consisted of 1.8 μL of the extracted DNA, 1.5 μL of each of the two universal primers, 10.2 μL of distilled water, and 15 μL of EmeraldAmp PCR Master Mix. The PCR amplification was performed at 94 °C for 2 min, followed by 36 cycles of 94 °C for 30 s, 45 °C for 1 min, and 72 °C for 1 min, and then followed by a final extension at 72 °C for 5 min. The PCR products were checked under UV transillumination after agarose gel electrophoresis, and then commercially sequenced using an automated sequencer in both directions using the LCO1490 and HCO2198 primers, respectively. Chromatograms were checked manually for misreads and then the sequences were trimmed in MEGA11 (Tamura et al. 2021). A total of 23 new COI sequences were subsequently uploaded and stored in GenBank under accession numbers: PQ145585–PQ145607 (Table 1).

**Table 1.** Information on the specimens used in this phylogenetic study.

Species/ specimen code	CUMZ code	Locality	GenBank accession number
**Family Veronicellidae Gray, 1840**			
*Valigunasiamensis* (Martens, 1867)			
Chai1NE-2	16043	Phakdi Chumphon, Chaiyaphum, Thailand	PQ145586
Chan6E-1	16052	Soi Dao, Chanthaburi, Thailand	PQ145587
Chan8E-2	16053	Makham, Chanthaburi, Thailand	PQ145585
CM7N-1	16017	Sa-moeng, Chiang Mai, Thailand	PQ145589
CP1S-1	16072	Pathio, Chumphon, Thailand	PQ145588
NB1NE-1	16031	Suwannakhuha, Nong Bua Lam Phu, Thailand	PQ145594
NKW4C-1	16037	Banphot Phisai, Nakhon Sawan, Thailand	PQ145598
NNY1E	16059	Ban Na, Nakhon Nayok, Thailand	PQ145596
Pat1S-1	16081	Mueang Pattani, Pattani, Thailand	PQ145595
Phet1C	16068	Nong Ya Plong, Phetchaburi, Thailand	PQ145593
Phet2C	16068	Nong Ya Plong, Phetchaburi, Thailand	PQ145592
Phet4C	16069	Kaeng Krachan Phetchaburi, Thailand	PQ145591
Phet5C	16069	Kaeng Krachan Phetchaburi, Thailand	PQ145590
TD2E-1	16058	Koh Kood, Trat, Thailand	PQ145597
MF983692	–	Bokpyin, Tanintharyi Region, Myanmar	MF983692
*Valigunasemicerina* sp. nov.			
Chan5E-2	16092	Khlung, Chanthaburi, Thailand	PQ145600
Chan5E-4	16092	Khlung, Chanthaburi, Thailand	PQ145601
Chon5E-1	16091	Sriracha, Chonburi, Thailand	PQ145606
TD2E-2	16093	Koh Kood, Trat, Thailand	PQ145602
TD2E-3	16093	Koh Kood, Trat, Thailand	PQ145607
TD4E	16087	Koh Kood, Trat, Thailand	PQ145603
SKW4E-2	16090	Khao Chakan, Sa Kaeo, Thailand	PQ145599
*Valigunacrispa* sp. nov.			
SR1S-1	16094	Ban Na San, Surat Thani, Thailand	PQ145604
SR1S-2	16097	Ban Na San, Surat Thani, Thailand	PQ145605
*Semperulawallacei* (Issel, 1874)			
DQ897673	–	Sabah, Malaysia	DQ897673
JX532109	–	Tutuila, American Samoa	JX532109
LC415571	–	Chichijima Island, Chichijima, Ogasawara, Tokyo, Japan	LC415571
LC415572	–	Miyako Islands, Taira, Miyakojima, Okinawa, Japan	LC415572
LC415573	–	Miyako Islands, Irabuikemasoe, Miyakojima, Okinawa, Japan	LC415573
LC415574	–	Okinawa Island, Naha, Okinawa, Japan	LC415574
LC636083	–	Naha, Okinawa, Japan	LC636083
LC636084	–	Naha, Okinawa, Japan	LC636084
LC636085	–	Miyakojima, Okinawa, Japan	LC636085
LC636086	–	Nakijin, Okinawa, Japan	LC636086
LC636087	–	Kume, Okinawa, Japan	LC636087
LC636088	–	Tonaki, Okinawa, Japan	LC636088
*Belocaulusangustipes* (Heynemann, 1885)			
KM489490	–	Horco Molle, Yerba Buena, Tucuman, Argentina	KM489490
*Latipeserinaceus* (Colosi, 1921)			
KM489478	–	Horco Molle, Yerba Buena, Tucuman, Argentina	KM489478
*Laevicaulisalte* (Férussac, 1822)			
KX514443	–	Selapadu, Guntur, Andhra Pradesh, India	KX514443
**Family Onchidiidae Rafinesque, 1815**			
*Onchidellafloridana* (Dall, 1885)			
HQ660035	–	Tobago	HQ660035
*Onchidiumvaigiense* Quoy & Gaimard, 1825			
HQ660040	–	Papua New Guinea	HQ660040

For mitochondrial phylogenetics, we chose four veronicellids [*Belocaulusangustipes* (Heynemann, 1885), *Latipeserinaceus* (Colosi, 1921), *Laevicaulisalte* (Férussac, 1822), and *Semperulawallacei* (Issel, 1874)] included as related taxa and two onchidiids [*Onchidellafloridana* (Dall, 1885) and *Onchidiumvaigiense* Quoy & Gaimard, 1825] for which sequences were available in GenBank as outgroups to root the phylogenetic tree. Genetic data of three *Semperula* species recorded in Thailand [*S.maculata* (Templeton, 1858), *S.birmanica* (Theobald, 1864), and *S.tailandensis*Thomé et al. 1994], one *Filicaulis* species recorded in Thailand [*Filicaulisbleekeri* (Keferstein, 1865)], and the other *Valiguna* species, *V.flava* (Heynemann, 1885) were not available in GenBank; therefore, they were not included in the phylogenetic analysis. Details of all the new COI sequences and other sequences downloaded from GenBank used in this analysis are shown in Table 1.

The COI sequences were aligned with MUSCLE, implemented in MEGA11 (Tamura et al. 2021). Mean genetic distances between and within species of *Valiguna*, as well as related species were calculated using the p-distance model implemented in MEGA11. Phylogenetic tree was constructed using the Bayesian inference (BI) method, through the CIPRES Science Gateway (Miller et al. 2010). Before the BI analysis, the dataset was divided into three partitions using Kakusan4 (Tanabe 2011) and the general time-reversible model (Tavaré 1986) with a gamma distribution being chosen for all three COI codon positions. The BI analysis was run in MrBayes on XSEDE v. 3.2.7a (Ronquist et al. 2012) using two simultaneous runs. The analysis was performed for 10,000,000 generations and sampled every 1,000 generations. The first 25% of the obtained trees were discarded as burn-in. Convergence of the two runs was completed when the average standard deviation (SD) values of split frequencies were less than 0.01. Posterior probabilities (PP) ≥ 0.95 were accepted as significant (San Mauro and Agorreta 2010).

Additionally, a maximum likelihood tree was generated using the IQ-TREE webserver, which includes the ModelFinder function (Nguyen et al. 2015; Trifinopoulos et al. 2016; Kalyaanamoorthy et al. 2017). Branch support was estimated with 10,000 ultra-fast bootstrap replicates (Hoang et al. 2018), the Shimodaira-Hasegawa approximate likelihood-ratio test (SH-aLRT), and the approximate Bayes test (aBayes) (Anisimova et al. 2011). SH-aLRT support values ≥ 80%, aBayes support values ≥ 0.95, and ultra-fast bootstrap support (BS) values ≥ 95% for each node were accepted as well supported (Anisimova et al. 2011; Hoang et al. 2018).

### ﻿Anatomical abbreviations

The following abbreviations were used in this study.

**ag** albumen gland

**an** anus

**bc** bursa copulatrix

**cj** canalis junctor

**db** duct of bursa copulatrix

**dd** distal posterior deferens

**fc** fertilisation complex

**fg** female genital pore

**gn** penis glans

**hg** hermaphroditic gland

**it** intermediate tubule

**lt** long tubule

**md** middle deferens

**mg** male genital pore

**ov** oviduct

**pa** papilla

**pb** penis base

**pd** proximal posterior deferens

**pk** peak; in whose extremity deferens opens

**pr** prostate gland

**re** rectum

**rm** retractor muscle

**sd** spermioduct

**st** short tubules

**sv** seminal vesicle

**vd** vas deferens

Institutional abbreviations

**CUMZ** Chulalongkorn University, Museum of Zoology, Bangkok, Thailand

## ﻿Results

### ﻿Molecular analysis

**Figure 2.**
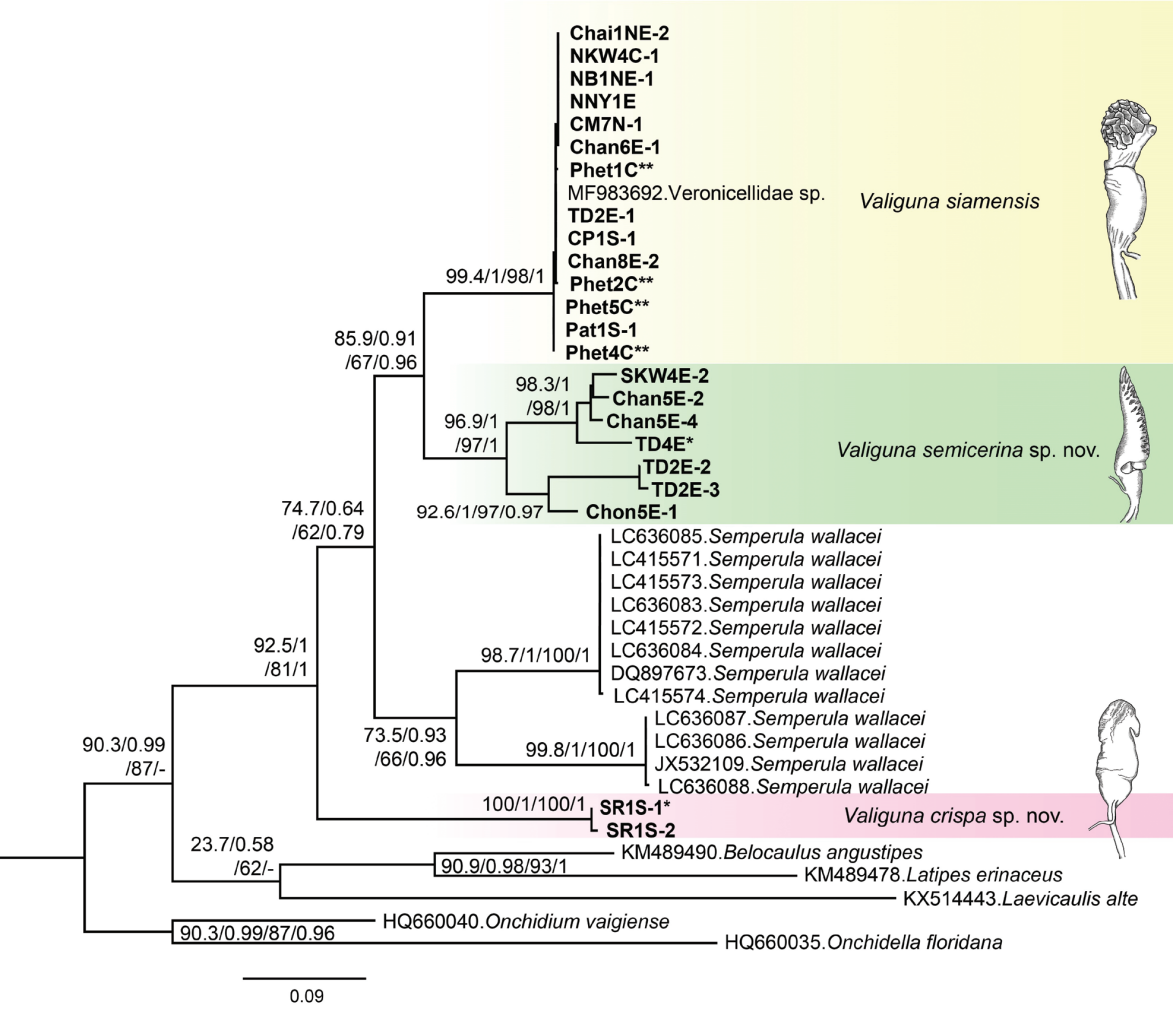
Phylogenetic tree showing the relationships among species of *Valiguna* based on the COI gene sequences. Numbers by the nodes are the ML BS (left) and BI PP (right) values. Note, SH-aLRT ≥ 80%, aBayes ≥ 0.95, BS ≥ 95%, and PP ≥ 0.95 for each node were determined to be well supported. (*) indicates the holotype and (**) indicates the topotype.

A total of 24 *Valiguna* sequences of COI (23 sequenced herein and one downloaded from GenBank), four veronicellid sequences (*Belocaulus* Hoffmann, 1925, *Latipes* Colosi, 1922, *Laevicaulis* Simroth, 1913, and *Semperula* Grimpe & Hoffmann, 1924), and two onchidiid sequences (*Onchidella* Gray, 1850 and *Onchidium* Buchannan, 1800) were used in the final analyses. The final COI alignment had a total length of 655 aligned nucleotides, containing 289 variable sites and 226 parsimony informative sites. Phylogenetic trees obtained from the COI dataset show that each species of *Valiguna* [*V.siamensis* (Martens, 1867), *V.semicerina* sp. nov., and *V.crispa* sp. nov.] was monophyletic with high support (Fig. 2). The *V.siamensis* was retrieved as the sister taxon to the *V.semicerina* sp. nov. The clade of *V.siamensis* + *V.semicerina* sp. nov. was grouped with the *S.wallacei* clade with low support (Fig. 2; SH-aLT = 74.7%, aBayes = 0.64, BS = 62%, PP = 0.79). The clade of *V.crispa* sp. nov. was sister to the *V.siamensis* + *V.semicerina* sp. nov. clade and *S.wallacei* clade (Fig. 2). In addition, in this study, both the BI and ML analyses did not resolve the phylogenetic relationships among five veronicellid genera (*Valiguna*, *Belocaulus*, *Latipes*, *Laevicaulis*, and *Semperula*) (Fig. 2).

The mean genetic distances of the COI gene observed among three species of *Valiguna*, as well as *Belocaulus*, *Laevicaulis*, *Latipes*, and *Semperula* ranged from 14.9% (*Valiguna* and *Semperula*) to 23.6% (*Laevicualis* and *Latipes*) (Table 2). Among *Valiguna* lineages, the average *p*-distances ranged from 13.5% (*V.siamensis* and *V.semicerina* sp. nov.) to 17.4% (*V.siamensis* and *V.crispa* sp.). The intraspecific genetic distances within the genus *Valiguna* ranged from 0.2% (*V.siamensis*) to 8.5% (*V.semicerina* sp. nov.) (Table 3).

**Table 2.** Pairwise genetic divergences among the *Belocaulus*, *Laevicaulis*, *Latipes*, *Onchidium*, *Onchidella*, *Semperula*, and *Valiguna* genera from the COI gene fragment sequences estimated by the *p*-distance model.

Genera	*Valiguna*	*Semperula*	*Laevicaulis*	*Belocaulus*	*Latipes*	*Onchidium*
***Valiguna***						
***Semperula***	0.149					
***Laevicaulis***	0.220	0.230				
***Belocaulus***	0.202	0.202	0.217			
***Latipes***	0.230	0.218	0.236	0.173		
***Onchidium***	0.191	0.184	0.219	0.203	0.221	
***Onchidella***	0.222	0.224	0.255	0.246	0.254	0.207

**Table 3.** Intraspecific and interspecific genetic distances of *Valiguna* spp.

Species	*V.siamensis*	*V.semicerina* sp. nov.	*V.crispa* sp. nov.
***V.siamensis***	0.002		
***V.semicerina* sp. nov.**	0.135	0.085	
***V.crispa* sp. nov.**	0.174	0.171	0.003

### ﻿Systematic accounts

**Family Veronicellidae Gray, 1840**

#### ﻿Genus Valiguna

Taxon classificationAnimaliaSystellommatophoraVeronicellidae

Grimpe & Hoffmann, 1925

E508E24F-C8C0-563E-8136-6BBA8D129E31

Semperula (Valiguna) Grimpe & Hoffmann, 1925a: 391, 392.
Valiguna : Hoffmann 1941: 236, 237. Gomes and Thomé 2004: 595, 596. Gomes 2007: 11, 12. Gomes et al. 2008: 163–169.

##### Type species.

*Vaginulaschneideri* Simroth, 1895, by original designation (Grimpe and Hoffmann 1925a: 392).

##### Diagnosis.

**Figure 3.**
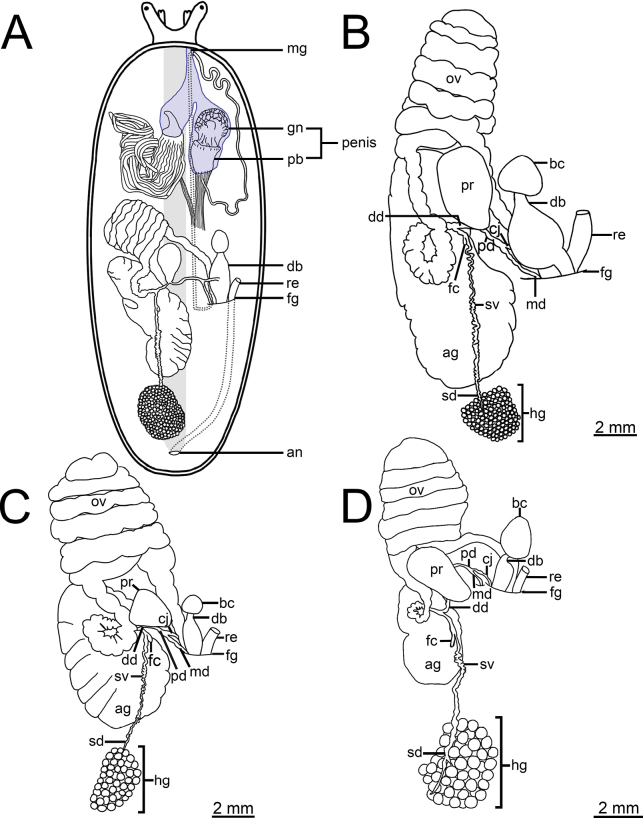
**A** schematic drawing showing an arrangement of the reproductive system of *Valiguna***B–D** female reproductive system of **B***V.siamensis*, specimen CUMZ 16054 from Chanthaburi **C***V.semicerina* sp. nov., holotype CUMZ 16087 from type locality, and **D***V.crispa* sp. nov., holotype CUMZ 16094 from type locality.

Male genitalia with penial retractor muscle attached to right hyponotum; penis and penial gland have separate muscular sheaths, fused anteriorly to form atrium that opens to male genital pore, and situated at base of lower right tentacle. Penis has penis base and penis glans, and an acropleurocaulis type of penis (vas deferens open at intermediate position between terminal and basal part of penis; Fig. 3A).

##### Remarks.

The nominal name *Valiguna* was initially proposed as a subgenus of *Semperula* to include a single species *Semperulaschneideri* (Simroth, 1895) from Sumatra (Grimpe and Hoffmann 1925a). Consecutively in the same year, this nominal genus-group name was disregarded and its type species was treated as *S.siamensisschneideri* (see Grimpe and Hoffmann 1925b; Hoffmann 1925). However, due to its unique penis shape and an acropleurocaulis type penis (vas deferens opens at the median of the penis), Hoffmann (1941) raised *Valiguna* to the genus level, recognised *Valigunaschneideri* as its valid species name again, and also introduced a second species, *V.isseli* Hoffmann, 1941 from Sarawak. Later, Gomes and Thomé (2004) and Gomes et al. (2008) reviewed the veronicellids slugs from Oriental and Australian regions based on the type specimens and additional materials. They transferred ‘*Vaginulaflava* Heynemann, 1885’ and ‘*Vaginulussiamensis* Martens, 1867’ to this genus and recognised ‘*V.schneideri*’ and ‘*V.isseli*’ as a synonyms of *V.flava*. Until now, only two species are validly recognised in this genus: *V.flava* is recorded from Sumatra and Borneo, and *V.siamensis* is widely distributed in Indochina and with a record from Sri Lanka (Gomes et al. 2008; Inkhavilay et al. 2019; Sutcharit et al. 2020a; GBIF 2023; MolluscaBase 2024).

#### ﻿ Valiguna siamensis

Taxon classificationAnimaliaSystellommatophoraVeronicellidae

(Martens, 1867)

B7A03219-AE5E-5FC6-8DAD-F869D8D60BA1

**Figure 4.**
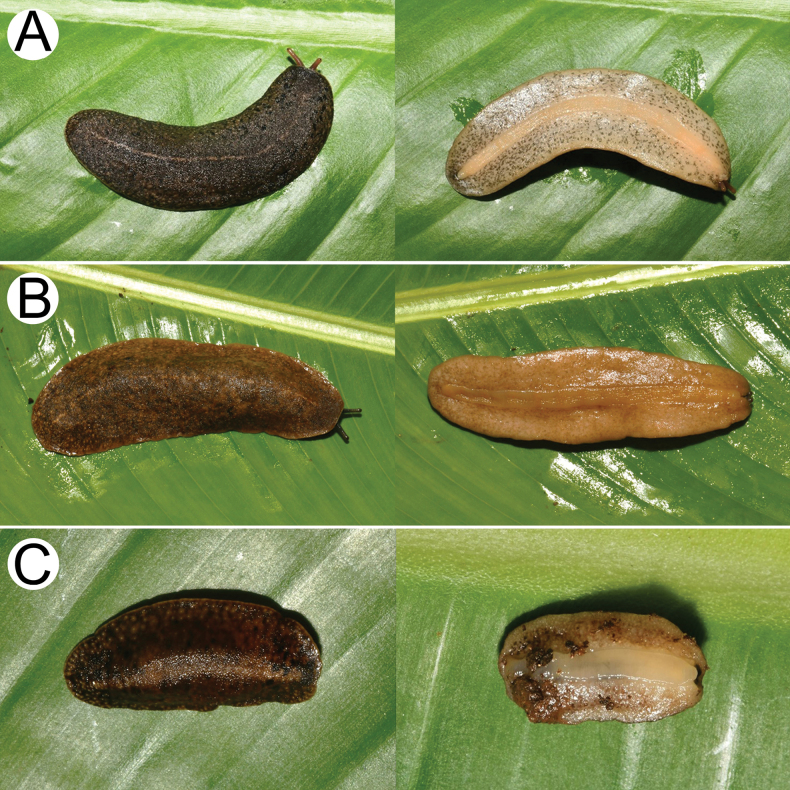
Living slugs **A***Valigunasiamensis*, specimen CUMZ 16038 from Khon Kaen (length ~ 60 mm) **B***V.semicerina* sp. nov., holotype CUMZ 16087 from type locality (length ~ 34 mm), and **C***V.crispa* sp. nov., holotype CUMZ 16094 from type locality (length ~ 28 mm). Dorsal view (left) and ventral view (right).

**Figure 5.**
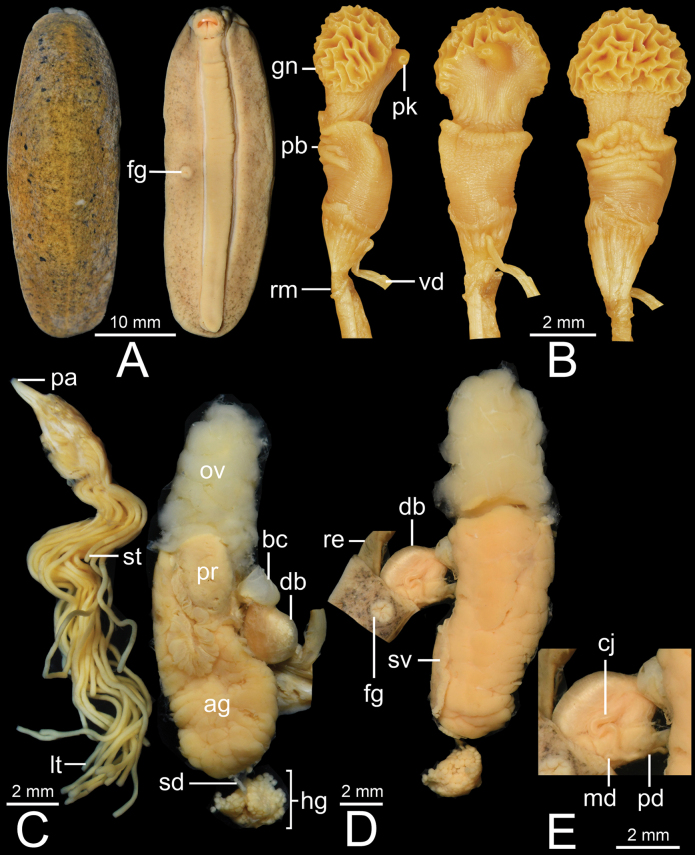
*Valigunasiamensis*CUMZ 16054 from Chanthaburi **A** dorsal and ventral views **B** penis **C** penial gland and penial tubules **D** dorsal and ventral views of female genitalia, and **E** inset of female genitalia.

**Figure 6.**
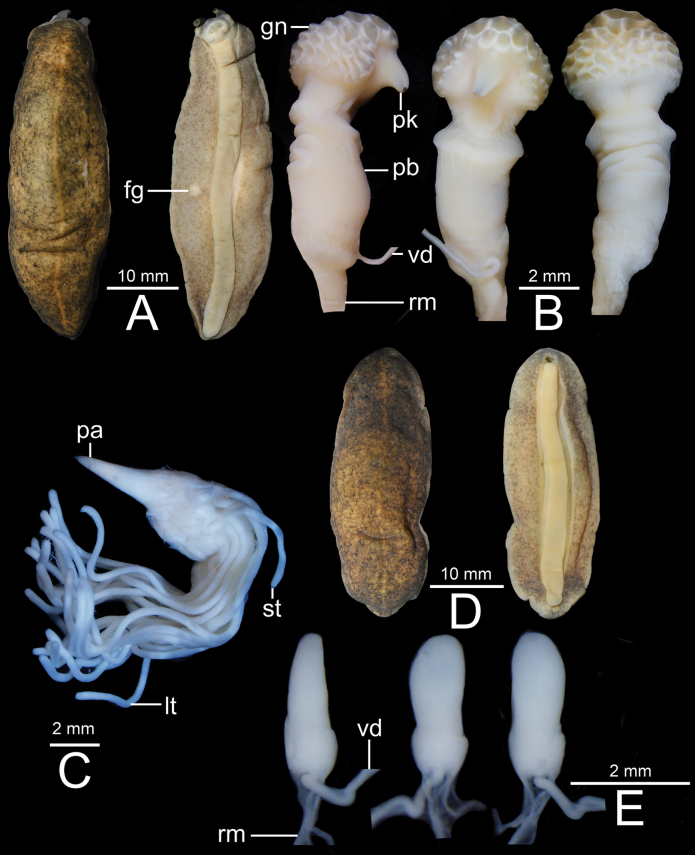
**A–C***Valigunasiamensis*, specimen CUMZ 16068 from Phetchaburi (topotype) and **D, E** specimen CUMZ 16060 from Bangkok. **A, D** dorsal and ventral views **B, E** penis of adult (**B**) and immature (**E**), and **C** penial gland and penial tubules.

**Figure 7.**
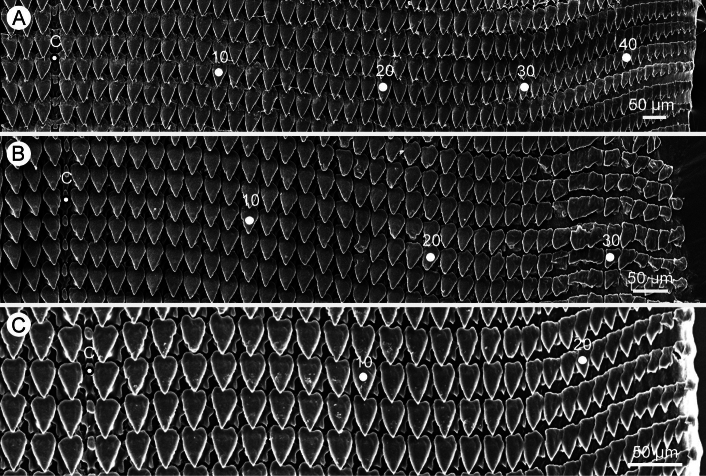
SEM images of radula **A***Valigunasiamensis*, specimen CUMZ 16054 from Chanthaburi **B***V.semicerina* sp. nov., holotype CUMZ 16087 from type locality, and **C***V.crispa* sp. nov., holotype CUMZ 16094 from type locality. Central tooth indicated by ‘C’.

Figs 2 , 3B , 4A , 5 , 6 , 7A

**Figure 8.**
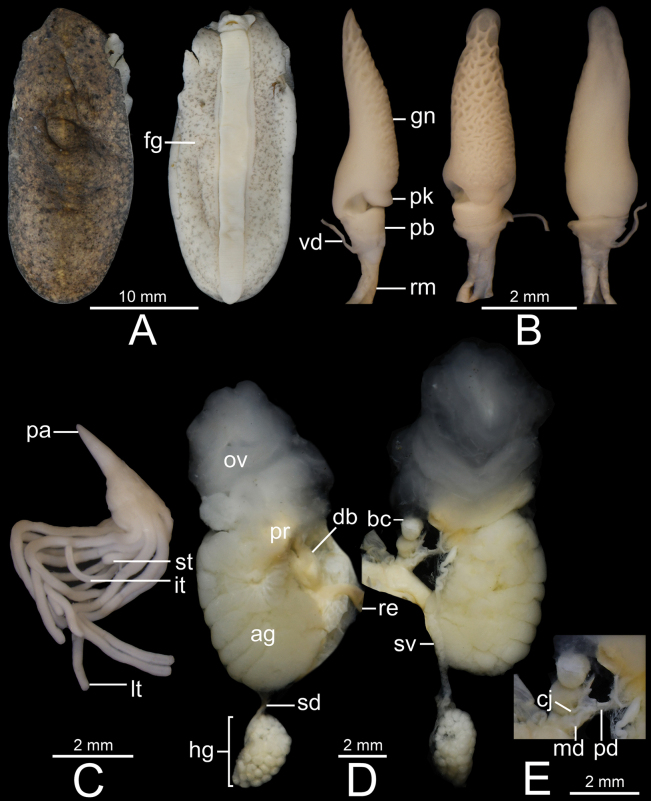
*Valigunasemicerina* sp. nov., holotype CUMZ 16087 from type locality **A** dorsal and ventral views **B** penis **C** penial gland and penial tubules **D** dorsal and ventral views of female genitalia, and **E** inset of female genitalia.

Vaginulus siamensis Martens, 1867: 68, pl. 5; fig. 3. Type locality: Petshaburi [Phetchaburi Province, Thailand].
Veronicella bocourti Rochebrune, 1882b: 117. Type locality: Bangkok [Thailand].
Veronicella chaudoensis Rochebrune, 1882a: 67. Type locality: Montagnes de Chadou [Chau Doc City, An Giang Province, Vietnam].
Veronicella titanotona Rochebrune, 1882a: 68. Type locality: Spiglumi-Britton [Cochinchina].
Vaginulus reticulatus Westerlund, 1883: 49. Type locality: Galle [Sri Lanka]. Thomé 1984: 29–32, figs 1–7.
Semperula bocourti : Grimpe and Hoffmann 1925b: 44. Hoffmann 1925: 181.
Semperula chaudoensis : Grimpe and Hoffmann 1925b: 44. Hoffmann 1925: 181.
Semperula titanotoma [sic]: Grimpe and Hoffmann 1925b: 44 [misspelling]. Hoffmann 1925: 181.
Semperula siamensis : Grimpe and Hoffmann 1925a: 388, 390–392. Grimpe and Hoffmann 1925b: 17–19, 44–47, fig. 8. Hoffmann 1925: 179–181, 256, 257, pl. 3, fig. 29, pl. 6, fig. 45k3, pl. 8, figs 58, 60, 61a, 62, pl. 9, fig. 64, pl. 10, fig. 71, pl. 11, figs 78–80. Solem, 1966: 21. Hemmen and Hemmen 2001: 40.
Valiguna siamensis : Gomes and Thomé 2004: 595, 596. Gomes et al. 2008: 164, 169, figs 13–18. Inkhavilay et al. 2019: 48, figs 19b, 55b. Sutcharit et al. 2020a: 16, 17, fig. 3d.

##### Material examined.

**Laos**: • Wat Oudom, Khamkeut, Bolikhamsai; 18°10'56.6"N, 104°59'04.6"E; CUMZ 16001. **Myanmar**: • Winner hotel, Kalaw, Shan; 20°37'40.4"N, 96°33'28.1"E; CUMZ 16002. **Thailand: Chiang Rai**: • Wat Tham Pha Mee, Mae Sai; 20°32'01.7"N, 99°52'34.5"E; CUMZ 16003; • Wat Tham Pha Chom, Mae Sai; 20°26'31.9"N, 99°52'23.5"E; CUMZ 16004; • Wat Pa Pha Mi, Mae Sai; 20°24'18.3"N, 99°51'09.9"E; CUMZ 16005; • Wat Tham Pum, Mae Sai; 20°20'54.1"N, 99°51'37.9"E; CUMZ 16006; • Wat Tham Pla Witthayakhom School, Mae Sai; 20°19'58.3"N, 99°51'52.9"E; CUMZ 16007; • Wat Tham Phra (Buddha Cave), Mueang Chiang Rai; 19°55'06.3"N, 99°47'19.5"E; CUMZ 16008; • Wat Tham Phra Pha Kok, Wiang Chai; 19°52'21.4"N, 100°02'41.9"E; CUMZ 16009; • Mae Lao Wildlife Breeding Center, Mae Lao; 19°45'57.6"N, 99°38'12.6"E; CUMZ 16010; • Wat Tham Phajarui, Pa Daet; 19°34'19.1"N, 99°59'17.3"E; CUMZ 16011; **Chiang Mai**: • Wat Tham Pha Phueng, Chai Prakan; 19°44'19.1"N, 99°05'16.2"E; CUMZ 16012; • Khun Mai Baan Suan Resort, Mae Ai; 20°03'46.7"N, 99°21'31.6"E; CUMZ 16013; • Wat Tham Tap Tao, Chai Prakan; 19°39'46.3"N, 99°07'03.4"E; CUMZ 16014; • Wat Tham Klaeb, Chiang Dao; 19°33'33.4"N, 99°03'46.9"E; CUMZ 16015; • Tham Chiang Dao, Chiang Dao; 19°23'38.3"N, 98°55'43.2"E; CUMZ 16016; • Homdoi Guesthouse, Samoeng; 18°51'04.5"N, 98°43'37.4"E; CUMZ 16017; • B-tel Chom Thong Resort, Chom Thong; 18°26'19.4"N, 98°40'20.9"E; CUMZ 16018; **Lampang**: • Tham Pha Thai, Ngao; 18°36'19.7"N, 99°53'51.9"E; CUMZ 16019; • Chao Por Pratu Pha Shrine, Mae Mo; 18°30'49.9"N, 99°49'09.9"E; CUMZ 16020; **Lamphun**: • Wat Pa Phai, Li; 17°52'22.6"N, 98°55'26.8"E; CUMZ 16021; • Wat Doi Daen, Ban Hong; 18°21'52.5"N, 98°45'42.3"E; CUMZ 16083; **Phrae**: • Huai Rong Waterfall, Rong Kwang; 18°26'31.6"N, 100°27'00.9"E; CUMZ 16022; **Bueng Kan**: • Wat Tham Pha Khao, Si Wilai; 18°11'27.3"N, 103°49'59.9"E; CUMZ 16023; • Wat Tham Phra Phu Wuua, Seka; 18°08'12.9"N, 103°59'34.8"E; CUMZ 16024; **Loei**: • Wat Tham Pha Baen, Chiang Khan; 17°54'24.6"N, 101°43'28.5"E; CUMZ 16025; • Wat Tham Pha Pu, Mueang Loei; 17°34'44.6"N, 101°42'40.0"E; CUMZ 16026; • Wat Thepnimitr, Phu Ruea; 17°28'03.3"N, 101°16'32.7"E; CUMZ 16027; • Wat Tham Erawan, Erawan; 17°20'43.5"N, 102°01'07.3"E; CUMZ 16028; • Wat Tham Pha Bing, Wang Saphung; 17°14'03.8"N, 101°44'14.6"E; CUMZ 16029; • Wat Tham Maholan, Nong Hin; 17°06'26.2"N, 101°52'43.7"E; CUMZ 16030; **Nong Bua Lam Phu**: • Wat Tham Suwannakhuha, Suwannakhuha; 17°36'29.2"N, 102°16'57.1"E; CUMZ 16031; • Wat Tham Pha Chor, Na Wang; 17°18'50.6"N, 102°07'05.0"E; CUMZ 16032; **Sakon Nakhon**: • Kham Hom Waterfall, Mueang Sakon Nakhon; 17°07'25.2"N, 104°01'07.3"E; CUMZ 16033; • Wat Tham Phu Pha Yon, Phu Phan; 16°56'28.4"N, 104°04'27.5"E; CUMZ 16034; **Tak**: • Taksin Maharat, Mueang Tak; 16°46'44.3"N, 98°55'42.7"E; CUMZ 16035; **Phitsanulok**: • Wat Ampharin Khuha (Tham Muang), Noen Maprang; 16°30'13.8"N, 100°41'20.9"E; CUMZ 16036; **Nakhon Sawan**: • Wat Thep Sathaporn, Banphot Phisai; 15°54'49.5"N, 99°53'03.9"E; CUMZ 16037; **Khon Kaen**: • Tham Phu Loop, Chum Phae; 16°49'48.6"N, 101°59'07.8"E; CUMZ 16038; • Khon Kaen University, Mueang Khon Kaen; 16°28'27.4"N, 102°49'18.9"E; CUMZ 16039; • Sawathi, Mueang Khon Kaen; CUMZ 16040; **Buriram**: • Socool Grand Hotel, Nang Rong; 14°38'12.5"N, 102°47'27.4"E; CUMZ 16041; • Wat Khao Phra Ang Khan, Chaloem Phra Kiat; 14°32'04.6"N, 102°50'04.3"E; CUMZ 16042; **Chaiyaphum**: • Tham Wua Daeng, Phakdi Chumphon; 16°04'35.2"N, 101°26'23.9"E; CUMZ 16043; **Nakhon Ratchasima**: • Wat Pa Mongkol Tham, Pak Thong Chai; 14°42'07.5"N, 101°47'12.7"E; CUMZ 16044; • Wat Tham Thep Nimit, Pak Chong; 14°36'14.4"N, 101°33'59.1"E; CUMZ 16045; • Soken Villa Khaoyai, Pak Chong; 14°32'06.7"N, 101°23'37.4"E; CUMZ 16046; **Sa Kaeo**: •Tham Khao Maka, Mueang Sa Kaeo; 13°47'16.5"N, 101°56'53.8"E; CUMZ 16047; • Wat Tham Thep Plub Plueng Thong, Wang Sombun; 13°26'56.9"N, 102°13'02.9"E; CUMZ 16048; • Tham Phet Pho Thong, Khlong Hat; 13°24'50.1"N, 102°19'31.3"E; CUMZ 16049; **Chon Buri**: • Wat Tham Khao Cha Ang On, Bo Thong; 13°12'33.5"N, 101°39'05.6"E; CUM 16050; • Wat Khao Cha Ang, Bo Thong; 13°12'00.9"N, 101°34'54.6"E; CUMZ 16051; **Chanthaburi**: • Khao Soi Dao Wildlife Sanctuary, Soi Dao; 13°06'15.0"N, 102°11'39.5"E; CUMZ 16052; • Palm plantation near Quan Yin shrine, Makham; 12°43'37.9"N, 102°08'12.7"E; CUMZ 16053; • Wat Laem Sadet, Tha Mai; 12°34'18.9"N, 101°53'26.3"E; CUMZ 16054; • Trok Nong Waterfall, Khlung; 12°32'39.6"N, 102°14'13.6"E; CUMZ 16055; **Trat**: • Trat 101 Hotel, Mueang Trat; 12°14'31.4"N, 102°30'40.4"E; CUMZ 16056; • Koh Chang, Koh Chang; 12°03'38.8"N, 102°19'37.1"E; CUMZ 16057; • Suanya Koh Kood resort & spa, Koh Kood; 11°39'58.9"N, 102°32'05.6"E; CUMZ 16058; **Nakhon Nayok**: • Ka Ang Waterfall, Ban Na; 14°20'35.0"N, 101°07'53.0"E; CUMZ 16059; **Bangkok**: • Chulalongkorn University, Pathum Wan; 13°44'15.5"N, 100°31'48.9"E; CUMZ 16060; **Kanchanaburi**: • Tham Khao Noi Bureau of Monks, Thong Pha Phum; 14°41'54.1"N, 98°31'31.4"E; CUMZ 16061; • Pilok, Thong Pha Phum; 14°40'16.3"N, 98°23'04.1"E; CUMZ 16086; • Tham Than Lot, Si Sawat; 14°40'17.3"N, 99°17'19.1"E; CUMZ 16062; • Wat Pa Tham Pha Dang, Thong Pha Phum; 14°38'43.6"N, 98°39'31.8"E; CUMZ 16063; • Srinakarin Dam, Si Sawat; 14°24'26.2"N, 99°07'33.7"E; CUMZ 16064; • Hellfire Pass, Sai Yok; 14°22'49.3"N, 98°55'50.2"E; CUMZ 16065; • Wat Pu Ta Khian, Sai Yok; 14°17'22.2"N, 99°00'28.9"E; CUMZ 16066; • Tham Krasae, Sai Yok; 14°06'18.8"N, 99°09'59.6"E; CUMZ 16067; **Phetchaburi**: • Wat Khao Bandai, Nong Ya Plong; 13°14'09.5"N, 99°41'21.3"E; CUMZ 16068; • Ban Aob Fha Resort, Kaeng Krachan; 12°53'55.4"N, 99°38'54.8"E; CUMZ 16069; **Prachuap Khiri Khan**: • Wat Tham Khao Mai Ruak, Thap Sakae; 11°25'43.0"N, 99°36'18.5"E; CUMZ 16070; **Chumphon**: • Tham Pisadan Monastery, Pathio; 10°45'35.6"N, 99°13'47.5"E; CUMZ 16071, 16072; • Tham Rub Ror, Tha Sae; 10°37'26.1"N, 99°06'47.2"E; CUMZ 16073; **Ranong**: • Tham Phra Khayang, Kra Buri; 10°19'36.6"N, 98°45'53.1"E; CUMZ 16074; • Baan Cueng Kao, Mueang Ranong; 9°56'36.4"N, 98°38'01.7"E; CUMZ 16084; **Surat Thani**: • Khao Sok Evergreen House, Phanom; 8°54'53.8"N, 98°32'43.0"E; CUMZ 16075; • Khao Sok River Home Resort, Phanom; 8°51'02.3"N, 98°42'21.3"E; CUMZ 16076; • Pa Dang, Chai Buri; 8°29'32.3"N, 98°58'39.0"E; CUMZ 16077; **Krabi**: • Wat Tham Prasat Nalakiring, Plai Phraya; 8°33'30.5"N, 98°51'44.1"E; CUMZ 16078; • Tham Khlang, Ao Luek; 8°20'17.8"N, 98°44'43.9"E; CUMZ 16079; **Trang**: • Wat Khao Lak Jan, Mueang Trang; 7°42'14.2"N, 99°43'46.3"E; CUMZ 16080; **Satun**: • Khao Toh Payawang, Mueang Satun; 6°37'39.4"N, 100°03'43.1"E; CUMZ 16085; **Pattani**: • Chabang Tiko, Mueang Pattani; 6°51'27.3"N, 101°15'12.2"E; CUMZ 16081, 16082.

##### Diagnosis.

Notum pale to dark brown with random tiny blackish spots and median dorsal line visible. Hyponotum and foot paler in colour, and with scattered pale blackish spots. Penis clavated with honeycomb-like glans; vas deferens opens at peak. Penial tubules consist of 19–29 long tubules and two short tubules.

##### External characteristics.

***Preserved specimen*.** Notum pale to dark brown colour with scattered tiny blackish spots. Body elongated ovate, 24–77 mm (mean 48.9 ± 10.0 mm) long and 9–28 mm (mean 14.8 ± 3.8 mm) wide. Some specimens with larger blackish spots scattered along left and right sides of notum. Median dorsal line thin and narrow, and a paler colour than notum. Hyponotum varies from pale yellow to beige or pale brown and with scattered tiny pale blackish spots. Foot pale yellow to beige, and narrower (width 2.0–7.0 mm, mean 3.4 ± 1.0 mm) than right hyponotum width. Male genital pore located closed to base of right lower-tentacle. Female genital pore located approximately midway of body on right hyponotum (Figs 5A, 6A, D).

***Live specimen*.** Notum pale to dark brown and with scattered tiny blackish spots. Median dorsal line thin and pale colour. Hyponotum varies from beige to pale brown colour, and with scattered pale blackish spots. Foot beige (Fig. 4A).

***Genital organs*.** Penis acropleurocaulis type; penis base (pb) cylindrical and relatively long. Penis glans (gn) starts from distal extremity of penis base, short cylindrical shaped, honeycomb formation at distal extremity, and bending forming pointed peak. Vas deferens (vd) opens at tip of peak. Peak (pk) cylindrical shaped and located at lateral side of penis glans (Figs 5B, 6B). Young specimens with smooth cylindrical penis (without honeycomb formation; Fig. 6E). Penial gland with conical papilla (pa). Penial tubules: 19–29 long tubules (lt) and two short tubules (st). Two long tubules appear distally bifurcated in some specimens (Figs 5C, 6C).

Bursa copulatrix duct (db) pyriform shaped, expanded in middle region, and then tapering to narrow tube on both ends. Bursa copulatrix (bc) almost circular shaped and connected to bursa copulatrix duct. Oviduct (ov) and albumen gland (ag) extremely enlarged, soft lobulated, and tufted; oviduct opened at female genital pore (fg). Distal (dd) and proximal (pd) posterior deferens inserted into somewhat oval-shaped prostate gland (pr). Middle deferens (md) penetrates into thick muscular right hyponotum then emerges near male genital pore (mg) before inserting into penis. Canalis junctor (cj) penetrated almost at middle of bursa copulatrix duct. Fertilisation complex (fc) short and small tube; seminal vesicle (sv) thin and curly, and attached to albumen gland with thin connective tissue. Spermioduct (sd) thin, unconvoluted, and contracts into numerous small globular hermaphroditic gland subunits. Hermaphroditic gland (hg) consists of many small subunits (Figs 3B, 5D, E).

***Radula*.** Teeth arranged in nearly straight rows, each row containing ~ 100 teeth with half row formula 1–49. Central teeth very small and unicuspid. Lateral and marginal teeth not differentiated, monocuspid, large triangular shaped, and with slightly pointed cusp. From inner to outer, lateromarginal teeth gradually narrower, smaller, and rather pointed cusp, and outermost teeth relatively small (Fig. 7A).

##### Distribution.

*Valigunasiamensis* occurs throughout Thailand and some have been reported from Cambodia, Laos, Myanmar, southern China, and Sri Lanka (Rochebrune 1882a, b; Gomes et al. 2008; Inkhavilay et al. 2019; Sutcharit et al. 2020a; GBIF 2023).

##### Comparative diagnosis.

*Valigunasiamensis* can be differentiated from *V.flava* in having a cylindrical penis with honeycomb-like glans, and with 21–31 unequal length (two short, 19–29 long) and two bifurcation penial tubules. In comparison, *V.flava* has a penis that is initially cylindrical, then tapers and bends with dentate and serrate glans. In addition, *V.flava* possesses only 15 short penial tubules (Table 4; Gomes et al. 2008).

**Table 4.** External and internal morphology of *Valiguna* species. Superscript number indicates the reference: ^1^Gomes et al. (2008).

Characters	*Valigunaflava* ^1^	*Valigunasiamensis*	*Valigunasemicerina* sp. nov.	*Valigunacrispa* sp. nov.
**Notum colour**	Pale yellowish brown to dark reddish brown with scattered wide blackish spots	Pale to dark brown with very tiny blackish spots scattered all around	Pale to dark brown with scattered tiny blackish spots	Dark brown with randomly scattered distinct, small, pale yellow and blackish spots all around
**Hyponotum colour**	Paler pale yellowish brown to dark reddish brown, without spots or only few tiny blackish spots	Beige to pale brown with many tiny pale blackish spots	White to pale beige or pale brown, without blackish spots or with many tiny spots	Beige with many tiny scattered blackish spots
**Teeth formula**	c/1+l46–47/2	c/1+l49/2	c/1+l31–35/2	c/1+l28/2
**Penial tubules**	15 short tubules	21–31 tubules: two short and 19–29 long (some are bifurcated)	18–22 tubules: four short, six intermediate, and 8–12 long (some long tubules are bifurcated)	11 long tubules
**Penis glans**	Cylindrical structure covered by dentate and serrate formations on dorsum of convex side	Short cylindrical with honeycomb structure at distal most end of glans	Elongated conical with honeycomb structure on one side and smooth surface on the opposite side	Short conical with wave-like structure
**Canalis junctor**	Penetrates bursa copulatrix duct near to the middle of the duct’s total length	Penetrates in bursa copulatrix duct near to the middle of the duct’s total length	Penetrates in bursa copulatrix duct near to the middle of the duct’s total length	Penetrates in bursa copulatrix duct near to the proximal end of the duct
**Bursa copulatrix**	Almost circular and concave in the middle	Almost circular	Almost circular	Almost oval
**Bursa copulatrix duct**	Cylindrical shaped	Pyriform and expanding at middle region	Short and narrow bottle-shaped duct near basal part of bursa copulatrix; looks similar to a bottle with neck positioned in the centre.	Short and narrow bottle-shaped duct near basal part of bursa copulatrix; looks similar to a bottle with neck positioned off-centre to one side.

**Figure 9.**
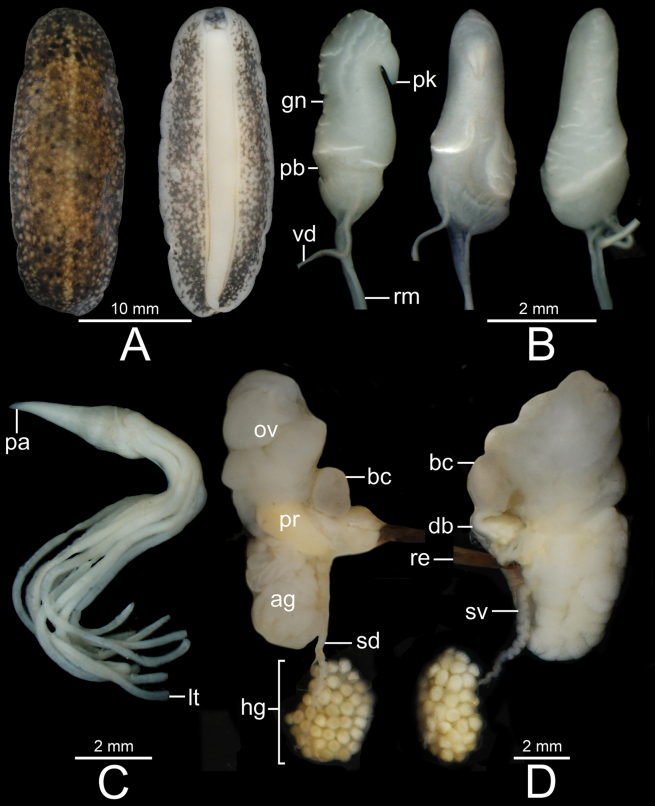
*Valigunacrispa* sp. nov., holotype CUMZ 16094 from type locality **A** dorsal and ventral views **B** penis **C** penial gland and penial tubules, and **D** dorsal and ventral view of female genitalia.

In addition, *Valigunasiamensis* also differs from *V.semicerina* sp. nov. and *V.crispa* sp. nov. in having their hermaphroditic glands composed of small-sized acini. In comparison, *V.semicerina* sp. nov. possesses hermaphroditic gland formed by medium-sized acini whereas *V.crispa* sp. nov. possesses hermaphroditic gland made up of large-sized acini (Figs 3B, C, D, 5D, 8D, 9D).

##### Remarks.

Originally, this species was nominated based on specimens from Phetchaburi Province, Thailand, with a brief description and illustrations. After examining the syntypes, Hoffmann (1925) relocated it to the *Semperula* and provisionally synonymised three poorly described species *Veronicellabocourti* Rochebrune, 1882, *Veronicellachaudoensis* Rochebrune, 1882, and *Veronicellatitanotona* Rochebrune, 1882 with this species. Later, Thomé (1984) examined the lectotype of *Vaginulusreticulatus* Westerlund, 1883 and recognised it as a synonym of *Semperulasiamensis*. Recently Gomes and Thomé (2004) transferred this species to the *Valiguna*, and Gomes et al. (2008) reported the genitalia based on specimens from Yunnan and Guangxi, China.

It is the predominant leatherleaf slug found in Thailand, occurring in diverse environments, including natural habitats as well as human-modified settings, such as residential areas, gardens, plantations, and greenhouses.

#### ﻿ Valiguna semicerina

Taxon classificationAnimaliaSystellommatophoraVeronicellidae

Mitchueachart & Panha sp. nov.

5285A6E4-6A17-5386-B168-1F8455986219

https://zoobank.org/04A64604-72E0-4A1B-87EE-797D056525B0

Figs 2 , 3C , 4B , 7B , 8

##### Type material.

***Holotype*** • CUMZ 16087 (length 34 mm, width 13 mm; Figs 4B, 8A). ***Paratype*** • CUMZ 16092 (2 specimens in ethanol).

##### Type locality.

Huang Nam Keaw Waterfall, Koh Kood, Trat Province, Thailand, 11°39'19.2"N, 102°34'53.3"E.

##### Other material examined.

**Cambodia**: • Recreational area near Sanker river, Traeng, Rotanak Mondol, Battambang Province; 12°49'51.4"N, 102°55'44.3"E; CUMZ 16088; **Myanmar**: • Dhammatat Cave, Mawlamyine Township, Mon State; 16°30'23.0"N, 97°48'36.3"E; CUMZ 16089; **Thailand: Sa Kaeo**: • Khao Chakan, Khao Chakan; 13°39'42.6"N, 102°05'22.5"E; CUMZ 16090; **Chonburi**: • Wat Khao Chalak, Sriracha; 13°11'49.3"N, 100°56'56.6"E; CUMZ 16091; **Trat**: • Suanya Koh Kood Resort & Spa, Koh Kood; 11°39'58.9"N, 102°32'05.6"E; CUMZ 16093.

##### Diagnosis.

Notum pale to dark brown with random tiny blackish spots, and median dorsal line visible. Hyponotum and foot paler in colour with or without scattered blackish spots. Penis rounded lanceolate with honeycomb-like glans on one side and smooth surface on opposite side; vas deferens opens at peak near basal part of honeycomb-like glans. Penial tubules consist of 18–22 tubules of varying lengths (short, intermediate, and long).

##### External characteristics.

***Preserved specimen*.** Notum pale to dark brown with scattered tiny blackish spots. Body elongated ovate, 32–45 mm (39.6 ± 5.1 mm) long, and 11–16 mm (13.4 ± 1.7 mm) wide. Median dorsal line thin and narrow, and paler colour than notum. Hyponotum white, beige to pale brown and with or without numerous tiny blackish spots. Foot white, pale yellow to beige, and narrower (width 2.0–4.0 mm) than right hyponotum width. Male genital pore located close to base of right lower tentacle. Female genital pore located approximately midway of body on right hyponotum (Fig. 8A).

***Live specimen*.** Notum pale to dark brown and with scattered tiny blackish spots. Median dorsal line thin and pale colour. Hyponotum varies from white to pale beige or pale brown, and with or without pale blackish spots. Foot white to pale beige (Fig. 4B).

***Genital organs*.** Penis acropleurocaulis type; penis base (pb) cylindrical and relatively short. Penis glans (gn) an elongated conical shape with honeycomb formation on peak side and smooth surface on opposite side. Vas deferens (vd) opens at tip of peak. Peak (pk) triangular shaped and located somewhat near penis base (Fig. 8B). Penial gland with conical papilla (pa). Penial tubules 18–22 tubules: comprised of four short tubules (st), six intermediate tubules (it), and 8–12 long tubules (lt); sometimes around five bifurcating tubules appeared on intermediate and long tubules (Fig. 8C).

Bursa copulatrix duct (db) bottle shaped, slightly enlarged cylindrical near genital pore and tapering to short and narrow near bursa copulatrix. Bursa copulatrix (bc) almost circular shaped and connected to bursa copulatrix duct. Oviduct (ov) and albumen gland (ag) extremely enlarged, soft lobulated and tufted; oviduct opened at female genital pore (fg). Distal (dd) and proximal (pd) posterior deferens inserted into triangular-shaped prostate gland (pr). Middle deferens (md) penetrates into thick muscular right hyponotum then emerges near male genital pore (mg) before inserting into penis. Canalis junctor (cj) penetrates almost at middle of bursa copulatrix duct. Fertilisation complex (fc) short and small tube; seminal vesicle (sv) thin and curly, and attached to albumen gland with thin connective tissue. Spermioduct (sd) thin, unconvoluted, and contracts into numerous small globular hermaphroditic gland subunits. Hermaphroditic gland (hg) consists of many medium-sized subunits (Figs 3C, 8D, E).

***Radula***. Teeth arranged in nearly straight rows, each row containing ~ 70 teeth with half row formula 1–35+. Central teeth very small and unicuspid. Lateral and marginal teeth not differentiated, monocuspid, large triangular shaped, and with slightly pointed cusp. From inner to outer, lateromarginal teeth gradually narrower, smaller, and rather pointed cusp, and outermost teeth relatively small and irregular shaped (Fig. 7B).

##### Etymology.

The specific name *semicerina* is from the Latin words *semis* meaning half and *cera* meaning honeycomb, which refers to the shape of the penis of this new species.

##### Distribution.

*Valigunasemicerina* sp. nov. is known to distributed in eastern Thailand, the Batttambang Province in Cambodia, and the Mon State in Myanmar (Fig. 1).

##### Comparative diagnosis.

*Valigunasemicerina* sp. nov. can be distinguished from *V.siamensis* and *V.flava* in having an elongated conical shape and honeycomb structure on peak side of penis glans surfaces, and with 18–22 unequal length (four short, six intermediate, and 8–12 long) and five bifurcation penial tubules. In comparison, *V.siamensis* possesses a cylindrical penis and honeycomb-like glans, and with 21–31 unequal length (two short and 19–29 long) and two bifurcation penial tubules, whereas *V.flava* possesses a cylindrical penis, tapering, and bends with dentate and serrate glans. Moreover, *V.flava* has 15 short penial tubules (Table 4; Gomes et al. 2008).

In addition, this new species also differs from *V.crispa* sp. nov. and *F.bleekeri* in having a honeycomb structure on penis glans, and with 18–22 unequal length and five bifurcation penial tubules. In comparison, *V.crispa* sp. nov. possesses a wavy-like structure on penis glans, and 11 equal length penial tubules without bifurcation (Table 4), whereas *F.bleekeri* possesses an acrocaulis cylindrical penis with submedial annular ridge, and with 12–18 short penial tubules (see Hoffmann 1925: pl. 5, fig. 45 c4).

##### Remarks.

This new species is superficially similar to *V.siamensis*. However, after examining lots of specimens of various sizes from several localities, *V.semicerina* sp. nov. generally has no tiny blackish spots on hyponotum, while *V.siamensis* tended to have many tiny pale blackish spots spread on the hyponotum.

#### ﻿ Valiguna crispa

Taxon classificationAnimaliaSystellommatophoraVeronicellidae

Mitchueachart & Panha sp. nov.

B6907F4F-CE1A-569A-9D36-7AB6E37348EC

https://zoobank.org/14A5F908-06EA-4E8C-82F6-859ECE3E1045

Figs 2 , 3D , 4C , 7C , 9

##### Type material.

***Holotype*** • CUMZ 16094 (length 28 mm, width 10 mm; Figs 4C, 9A). ***Paratype*** • CUMZ 16097 (1 specimen in ethanol).

##### Type locality.

Tham Kamin, Ban Na San, Surat Thani Province, Thailand, 8°49'49.4"N, 99°22'44.2"E.

##### Diagnosis.

Notum dark brown with random pale yellow and blackish spots, and median dorsal line visible. Hyponotum and foot paler in colour, and with scattered blackish spots. Penis cylindrical shaped and distally curved with wavy-like glans; vas deferens opens at peak. Penial tubules consist of 11 long tubules.

##### External characteristics.

***Preserved specimen*.** Notum pale brown with scattered small pale yellow and blackish spots. Body elongated ovate, 21–28 mm (24.5 ± 3.5 mm) long and 8–10 mm (9.0 ± 1.0 mm) wide. Median dorsal line thin and narrow, and paler colour than notum. Hyponotum pale beige and with many tiny blackish spots. Foot beige, and narrower (width 2.0 mm, mean 2.0 ± 0.0 mm) than right hyponotum width. Male genital pore located close to base of right lower tentacle. Female genital pore located approximately midway of body on right hyponotum (Fig. 9A).

***Live specimen*.** Notum dark brown with scattered tiny pale beige and blackish spots. Median dorsal line thin and indistinct colour. Hyponotum beige with scattered tiny blackish spots. Foot beige (Fig. 4C).

***Genital organs*.** Penis acropleurocaulis type; penis base (pb) slightly concave, surrounding penis and relatively short. Penis glans (gn) elongated conical shaped, slightly tapering, and then bending to form pointed peak with wavy-like structure on penis glans surface. Vas deferens (vd) opens at tip of peak. Peak (pk) cylindrical shaped and located at lateral side of penis glans (Fig. 9B). Penial gland with conical papilla (pa). Penial tubules with 11 long tubules (lt) (Fig. 9C).

Bursa copulatrix duct (db) bottle shaped, slightly enlarged cylindrical near genital pore and tapering to short and narrow near bursa copulatrix; looks similar to a bottle with neck positioned off-centre to one side. Bursa copulatrix (bc) almost oval shaped and connected to bursa copulatrix duct. Oviduct (ov) and albumen gland (ag) enlarged, soft lobulated, and tufted; oviduct opened at female genital pore (fg). Distal (dd) and proximal (pd) posterior deferens inserted into somewhat oval-shaped prostate gland (pr). Middle deferens (md) penetrates into thick muscular right hyponotum then emerges near male genital pore (mg) before inserting into penis. Canalis junctor (cj) penetrates at nearly proximal of bursa copulatrix duct (near female genital pore). Fertilisation complex (fc) short and small tube; seminal vesicle (sv) thin and curly, and attached to albumen gland with thin connective tissue. Spermioduct (sd) thin, unconvoluted, and contracts into numerous globular hermaphroditic gland subunits. Hermaphroditic gland (hg) consists of many large subunits (Figs 3D, 9D).

***Radula***. Teeth arranged in nearly straight rows, each row containing 57 teeth with half row formula 1–28. Central teeth very small and unicuspid. Lateral and marginal teeth not differentiated, monocuspid, large triangular shaped, and with slightly pointed cusp. From inner to outer, lateromarginal teeth gradually narrower, smaller, and rather pointed cusp, and outermost teeth relatively small (Fig. 7C).

##### Etymology.

The specific name *crispa* is from the Latin word *crispus* meaning wavy or curly, which refers to the structure found on the penis surface of this species.

##### Distribution.

*Valigunacrispa* sp. nov. is known only from the type locality, a limestone outcrop with low vegetation in southern Thailand (Fig. 1).

##### Comparative diagnosis.

*Valigunacrispa* sp. nov. differs from *V.siamensis* by having a wavy-like structure on penis glans, and with 11 tubules of equal length without any bifurcation, whereas *V.siamensis* possesses a cylindrical-shaped honeycomb structure at the tip of penis glans, along with 21–31 penial tubules of varying lengths (two short and 19–29 long), and two bifurcation penial tubules (Table 4).

This new species can also be distinguished from *V.flava* by having wavy structure on penis glans, and with 11 tubules of equal length without any bifurcation. In comparison, *V.flava* presents a surrounding structure at base of penis glans, resembling annular ring, and glans has dentate and serrate formations that envelop the dorsal side of the glans curvature. Additionally, *V.flava* possesses approximately 15 short penial tubules (Table 4; Gomes et al. 2008).

##### Remarks.

Generally, *V.crispa* sp. nov. has an external morphology very similar to the other congeners, except that this new species tended to have a weaker or indistinct median dorsal line.

## ﻿Discussion

Prior to this study, twelve species of veronicelloidid slugs were recognised in Thailand, comprised of seven species of the rathouisiid carnivorous slugs in the genus *Atopos* Simroth, 1891, and five species of the veronicellid leatherleaf slugs: three species of *Semperula*, one species of *Filicaulis*, and one species of *Valiguna* (Collinge 1902, 1903; Grimpe and Hoffmann 1925a, 1925b; Hoffmann 1925; Hemmen and Hemmen 2001; Maassen 2001; Gomes and Thomé 2004). In this study, we present the diversity of the *Valiguna* in Thailand as being increased to three species based on combined molecular and morphological information. There is the previously known species, *V.siamensis* which is widely distributed in mainland Southeast Asia, plus two new species described herein as *V.crispa* sp. nov., which is endemic to southern Thailand, and *V.semicerina* sp. nov., which occurs in Thailand, Cambodia, and Myanmar.

Although the three *Valiguna* species recognised in this study superficially exhibit a similar external morphology, some characteristics of the hyponotum and notum can be tentatively used for species identification after examining numerous specimens that vary in size, as supported by the genitalia and DNA data. These three *Valiguna* species have clearly distinct morphologies of their penis glans and bursa copulatrix duct and have high inter-specific genetic divergences. Based on our observations, *V.siamensis* displays numerous tiny blackish spots scattered across the hyponotum and has a distinct median dorsal line. In contrast, *V.crispa* sp. nov. tends to have an indistinct median dorsal line; and *V.semicerina* sp. nov. has a generally uniformly coloured hyponotum (without spots). Therefore, the presence of the blackish spots on the hyponotum and median dorsal line (on notum) can be used to distinguish all three *Valiguna* species in Thailand.

The phylogenetic relationships within the *Valiguna* were investigated in order to verify their phylogenetic position and taxonomic validity, especially the most widespread species *V.siamensis*. Our results strongly confirm the monophyly of each *Valiguna* species. Nevertheless, *Valiguna* was placed as a sister clade to *Semperula* but with low support and the relationships among the two genera remain unresolved. The descriptions of the vas deferens opening position in *Semperula* are still uncertain [e.g., sub-basal to sub-terminal opening (Grimpe and Hoffmann 1924), lateral opening (Grimpe and Hoffmann 1925a; Hoffmann 1925), or basal opening (Grimpe and Hoffmann 1925b; Gomes and Thomé 2004; Gomes 2007; Gomes et al. 2008)]. Future studies should encompass additional genes and a larger number of veronicellid specimens to enhance our understanding of the phylogenetic relationships and morphological evolution among these veronicellids.

The widespread species, *V.siamensis*, exhibited a low genetic divergence among different geographical populations (mean genetic distance = 0.2%). In a similar case, the two widespread Southeast Asian snails species *Sarikasiamensis* (Pfeiffer, 1856) and *S.resplendens* (Philippi, 1847) from Thailand, showed low levels of genetic differentiation in their mitochondrial COI sequences (Dumidae et al. 2020; Pholyotha et al. 2020b, 2022). Moreover, a low 16S rRNA genetic diversity of an invasive species, the giant African snail *Lissachatinafulica* (Bowdich, 1822), across Thailand has also been reported (Dumidae et al. 2021). All these widespread species have successfully survived in both natural forest and anthropogenic habitats and play crucial roles as agricultural pests, while they have been documented as natural intermediate hosts for the rat lungworm that causes eosinophilic meningitis in humans in Thailand (Vitta et al. 2016).

In contrast, the phylogenetic tree indicates a high genetic divergence among populations of *V.semicerina* sp. nov. (mean genetic distance = 8.5%), even though they occur in nearby areas. For example, between *V.semicerina* sp. nov. specimens TD4E and TD2E that were collected from different localities in Koh Kood, a small island in the Gulf of Thailand. However, the sequence of TD4E was grouped with Chan5E from Chanthaburi and SKW4E from Sa Kaeo. In contrast, the sequence from TD2E was grouped with Chon5E from Chonburi (Figs 1, 2). The genetic divergence for the mitochondrial COI sequences in other land snails in mainland Southeast Asia, especially Thailand, previously revealed that interspecific distances in the systellommatophoran genera varied by 6.5–12.7% amongst *Phyllocaulis* in the Veronicellidae (Gomes et al. 2010) and by 3.2–15.7% amongst *Peronia* in the Onchidiidae (Dayrat et al. 2020). Furthermore, interspecific distances in the stylommatophoran genera varied from 2.7% to 13.7%, such as *Sarika* (5.2–13.0%; Pholyotha et al. 2022), *Taphrenalla* (3.4–7.7%; Pholyotha et al. 2020a), *Sophina* (4.5–13.7%; Sutcharit et al. 2020b), *Aenigmatoconcha* (9.7–12.0%; Pholyotha et al. 2021), and *Siamoconus* (2.7–4.0%; Pholyotha et al. 2023); while genetic divergences among lineages of the basommatophoran genus *Biomphalaria* varied by 1–12% (Palasio et al. 2017), and by 3.3–19.3% among lineages of the caenogastropod genus *Rhiostoma* (Tongkerd et al. 2023). In this study, the lack of distinct morphological features between populations of *V.semicerina* sp. nov. is a challenging problem for taxonomy despite their genetic distinctiveness, and it suggests that there are still cryptic species within this species.

This research expands the knowledge of the Siamese leatherleaf slugs in the Veronicellidae. It suggests that further investigation of the morphological and genetic diversity of Southeast Asian leatherleaf slugs is still required to provide a comprehensive species list to guide efforts in conservation and resource management.

## Supplementary Material

XML Treatment for Valiguna

XML Treatment for Valiguna siamensis

XML Treatment for Valiguna semicerina

XML Treatment for Valiguna crispa

## ﻿Additional information

### Author ORCIDs

Chirasak Sutcharit https://orcid.org/0000-0001-7670-9540

### Data availability

All of the data that support the findings of this study are available in the main text.
